# Virtual tumor boards: An approach to equity in cancer care

**DOI:** 10.34172/hpp.2023.23

**Published:** 2023-09-11

**Authors:** Mina AkbariRad, Sareh Keshvardoost, Hanie Shariatmadari, Abdollah Firoozi, AmirAli Moodi Ghalibaf

**Affiliations:** ^1^Department of Internal Medicine, Faculty of Medicine, Mashhad University of Medical Sciences, Mashhad, Iran; ^2^Cancer Research Center, Mashhad University of Medical Science, Mashhad, Iran; ^3^Faculty of Pharmacy, Mashhad University of Medical Sciences, Mashhad, Iran; ^4^Student Research Committee, Faculty of Medicine, Birjand University of Medical Sciences, Birjand, Iran; ^5^Student Committee of Medical Education Development, Education Development Center, Birjand University of Medical Sciences, Birjand, Iran

## To Editor,

 Cancer continues to be a major global health challenge, with low- and middle-income countries (LMICs) bearing a disproportionate burden of the disease. By 2030, nearly three-quarters of all cancer deaths are projected to occur in LMICs, underscoring the urgent need for effective strategies to bridge the gap between developed and non-developed countries.^1^ A multidisciplinary approach involving oncologists, radiotherapists, surgeons, and pathologists is crucial for optimal cancer management and treatment decision-making. Tumor board meetings, where experts convene to discuss treatment options, have proven instrumental in achieving accurate diagnoses, informed treatment choices, and improved patient outcomes.^2^

 However, the accessibility of tumor board meetings remains a significant challenge, particularly for patients in remote areas who face limited access to specialized cancer treatments, transportation barriers, and financial constraints. Furthermore, even within specialized centers, disagreements between experts during face-to-face tumor board meetings can hinder decision-making. This is where digital technologies, exemplified by the increased use of telemedicine during the COVID-19 era, offer a promising solution. By conducting tumor board meetings virtually, healthcare professionals in underprivileged areas can remotely consult with experts from specialized centers, facilitating evidence-based decision-making, fostering collaboration between health centers, optimizing the allocation of healthcare resources, and reducing the associated costs and time burden for patients and professionals.

 To illustrate the potential of virtual tumor boards, a study conducted in western Kenya implemented teleconsultation between a treating surgeon and three neurosurgeons from the United States of America using a web-based platform. The study successfully guided the treatment path of a 5-year-old child diagnosed with a challenging intracranial tumor with symptomatic hydrocephalus.^3^ Similarly, Mainguene et al examined the feasibility and effectiveness of virtual tumor boards, finding that they improved the accuracy of treatment decision-making for patients with advanced lung cancer and significantly reduced the time required to initiate treatment.^4^ Another study by Martiniuk et al explored the use of digital resources and virtual tumor boards for multidisciplinary discussions in pediatric oncology cases in rural and other low-resource settings, demonstrating that they led to improved clinical decision-making and increased the likelihood of appropriate treatment recommendations for pediatric cancer patients.^5^

 Additionally, Davis et al compared online and face-to-face tumor boards in the United States of America, reporting a 46% increase in doctor attendance during virtual meetings.^6^ Similarly, Hammer and colleagues’ study in an academic healthcare cancer center in the United States demonstrated that a virtual ear, nose, and throat tumor board reduced the delay time for case discussions from 23% to 10%.^7^ Another study by Irwin et al evaluated the impact of a virtual tumor board on the management of cancer, showing that the virtual interventions address cancer care barriers for marginalized populations via collaborative models, leveraging technology, community partnerships, and addressing multi-level barriers. Cultivating trust and collaboration are key for equitable oncology advancement.^8^

 Furthermore, a study conducted in Germany by Heuser et al examined the implementation of a virtual tumor board in a comprehensive cancer center. The study found that virtual tumor boards improved interdisciplinary communication, reduced the time to treatment decision, and facilitated the integration of precision medicine approaches.^9^ Similarly, a study by Esteso et al evaluated the use of virtual tumor boards in gastrointestinal oncology, claiming multidisciplinary tumor boards enhance cancer care, reducing disparities and costs, benefiting patients, and health systems.^10^ Moreover, Gebbia et al explored the implementation of virtual tumor boards for Lung cancer and demonstrated benefits include extended advantages of traditional MTB. Virtualization enhances participation, aids patient safety, promotes multidisciplinary interaction, and supports specialized treatment decisions in biomarker-driven strategies.^11^

 While online tumor boards offer substantial benefits for patients in remote regions and serve as valuable training tools, they also present certain challenges. One of the key issues is the requirement for internet access and a suitable platform for participation. Ensuring the active participation and accountability of all team members throughout the meeting while maintaining secure communication platforms is another critical consideration.

 Addressing these challenges necessitates the development of appropriate infrastructures, such as broadband networks and secure data transfer protocols. Furthermore, allocating funds to training and educational programs dedicated to the use of online tumor boards, establishing clear guidelines for their implementation, encouraging healthcare professionals to participate, simplifying health platforms for ease of use, accessibility, and security are essential steps toward maximizing the benefits of virtual tumor boards. Additionally, timely provision of patient information before meetings is vital to ensure adequate preparation and active engagement of all team members.

## Conclusion

 In conclusion, virtual tumor boards hold significant potential for improving cancer care, particularly for patients in remote regions, and can serve as valuable tools for physicians, especially in developing countries. However, overcoming challenges related to internet access, platform selection, and maintaining active participation throughout the meeting is critical. By investing in necessary infrastructures, training programs, and setting clear guidelines, healthcare systems can leverage the benefits of virtual tumor boards to enhance equity in cancer care.

## Competing Interests

 The authors have no competing interests to declare.

## Data Availability Statement

 Not applicable.

## Ethical Approval

 Not applicable.

## Funding

 Nil.
